# MaOpy2, a Transmembrane Protein, Is Involved in Stress Tolerances and Pathogenicity and Negatively Regulates Conidial Yield by Shifting the Conidiation Pattern in *Metarhizium acridum*

**DOI:** 10.3390/jof8060587

**Published:** 2022-05-30

**Authors:** Zhiqiong Wen, Yu Fan, Yuxian Xia, Kai Jin

**Affiliations:** 1 Genetic Engineering Research Center, School of Life Sciences, Chongqing University, Chongqing 401331, China; 201726021012@cqu.edu.cn (Z.W.); 201926021012@cqu.edu.cn (Y.F.); 2 Chongqing Engineering Research Center for Fungal Insecticide, Chongqing 401331, China; 3 Key Laboratory of Gene Function and Regulation Technologies Under Chongqing Municipal Education Commission, Chongqing 401331, China

**Keywords:** *Megatherium acridum*, Opy2, stress tolerance, virulence, conidiation pattern

## Abstract

Opy2 is an important membrane-anchored protein upstream of the HOG-MAPK signaling pathway and plays important roles in both the HOG-MAPK and Fus3/Kss1 MAPK. In this study, the roles of *MaOpy2* in *Metarhizium acridum* were systematically elucidated. The results showed that the *MaOpy2* disruption significantly reduced fungal tolerances to UV, heat shock and cell-wall-disrupting agents. Bioassays showed that the decreased fungal pathogenicity by topical inoculation mainly resulted from the impaired penetration ability. However, the growth ability of ∆*MaOpy2* was enhanced in insect hemolymph. Importantly, *MaOpy2* deletion could significantly increase the conidial yield of *M. acridum* by shifting the conidiation pattern from normal conidiation to microcycle conidiation on the 1/4SDAY medium. Sixty-two differentially expressed genes (DEGs) during the conidiation pattern shift, including 37 up-regulated genes and 25 down-regulated genes in ∆*MaOpy2*, were identified by RNA-seq. Further analysis revealed that some DEGs were related to conidiation and hyphal development. This study will provide not only the theoretical basis for elucidating the regulation mechanism for improving the conidial yield and quality in *M. acridum* but also theoretical guidance for the molecular improvement of entomopathogenic fungi.

## 1. Introduction

Entomopathogenic fungi can penetrate directly into the hemocoel of the host to utilize its nutrition [1], which offers great potential for insect pest control in the insect population and a low possibility of inducing the resistance of insects [2,3,4]. However, some disadvantages have limited their wide application, such as the long time required to kill insect pests, their sensitivity to diverse environmental conditions and the high cost for production [5,6,7]. As the main infective unit of entomopathogenic fungi and the active components of mycopesticides, conidia can tolerate various types of environmental stress and lead to an epizootic development in the insect populations [8]. Therefore, the conidial quality and yield of entomopathogenic fungi directly determine the efficiency of mycopesticides and the production cost. Elucidating the regulatory mechanism for improving the conidial yield and quality (stress tolerance, virulence, etc.) is expected to provide a theoretical basis which will promote the development of mycopesticides.

Two conidiation patterns, the normal conidiation pattern and the microcycle conidaition pattern, are found in most filamentous fungi [9,10]. Normal conidiation must go through a period of hyphae elongation and then form conidiophores at the tip of the long hyphae; however, microcycle conidiation can bypass the long hyphae growth and produce conidia directly [10]. These two conidiation patterns could be interconvertible under some specific circumstances [11,12,13,14]. In the locust-specific pathogenic fungus, *Metarhizium acridum*, microcycle conidiation exhibited a greater potential in large-scale applications than normal conidiation [15]. However, the mechanism of this shift remains unclear. Therefore, elucidating the underlying mechanism(s) of the conidiation pattern shift in order to improve the conidial productivity and quality is of great importance in entomopathogenic fungi.

As a cell transmembrane protein in fungi, *Opy2* plays distinct roles in different species. In *Saccharomyces cerevisiae*, Opy2 participates in the SHO1 branch of the high osmolarity glycerol (HOG) pathway and is identified as an osmosensor interacting with Ste50 [16]. In *Candida albicans*, *CaOpy2* plays a crucial role in cell wall stress tolerance and is essential for the phosphorylation of Cek1 [17]. In *Metarhizium robertsii*, an alternative transcription start site is achieved, and the regulation of the *MrOpy2* transcription level affects the fungal lifestyle and contributes to virulence [18]. In *Magnaporthe oryzae*, *MoOpy2* plays essential roles in pathogenicity, turgor pressure, appressorium formation, conidiation and hyphal growth [19]. However, in the entomopathogenic fungus *M. acridum*, the biological functions of *MaOpy2* have not been systematically elucidated.

Here, the functions of *Opy2* were identified in *M. acridum*. It was found that *MaOpy2* plays important roles in fungal growth, stress tolerance and fungal pathogenicity in *M. acridum*. Importantly, we present that *MaOpy2* disruption resulted in an increased conidial yield through the regulation of the conidiation pattern shift. RNA-seq showed that *MaOpy2* governs the conidiation pattern shift by regulating some gene expressions related to hyphae growth and conidiation in *M. acridum*.

## 2. Materials and Methods

### 2.1. Strains and Vector Construction

For gene deletion construction, the wild type (WT) strain *CQMa102* was cultured on 1/4 SDAY (1/4 Sabouraud dextrose agar plus yeast, 10 g glucose, 2.5 g yeast extract, 2.5 g peptone and 18 g agar per liter) for 15 days. *Escherichia coli* DH5α (Bioground Biotech, Beijing, China) and *Agrobacterium tumefaciens* AGL1 (Weidi Biotechnology, Shanghai, China) were adopted for vector construction and fungal transformation, respectively. For *MaOpy2* deletion vector construction, the fragments of the up- and downstream of *MaOpy2* were amplified with the primer pairs *Opy2-LF/Opy2-LR* and *Opy2-RF/Opy2-RR*, which were clonedinto *pK2-SM-F* and *pK2-SM-R* to form *pK2-SM-MaOpy2-F* and *pK2-SM-MaOpy2-R*. The 5′-flanking region and *MaOpy2* coding region (3397 bp) and downstream sequence (1398 bp) were cloned into the plasmid pK2-Nat to yield the *pK2-MaOpy2-Nat* vector, which was used for *MaOpy2* complementation (which was resistant to 75 μg/mL nourseothricin sulfate (Harveybio, Beijing, China)). Fungal transformation and transformant validation were conducted as reported previously [20]. The primers used in this work are listed in Table S1.

### 2.2. Phenotypic and Pathogenicity Analyses

Conidial germination and conidial yield assays were conducted as described previously [21]. Briefly, the conidial suspensions (1 × 10^7^ conidia/mL) of fungal strains were evenly spread on a 1/4 SDAY medium, and every 2 h, conidial germination was recorded. For the conidial yield, 2 μL suspensions (1 × 10^7^ conidia/mL) were spotted onto 24-well plates, and every 3 days, conidial yields were calculated. Additionally, for fungal sensitivity to heat shock, conidial suspensions (1 × 10^7^ conidia/mL) treated with a water bath at 45 °C for 3 h, 6 h, 9 h and 12 h were spread on a 1/4 SDAY medium and then cultured at 28 °C for 20 h to access the conidial germination rates. Similarly, for the UV-B tolerance assay, the conidial suspensions (1 × 10^7^ conidia/mL) were spread on 1/4 SDAY and exposed to UV-B radiance with a dose of 1350 mW/m^2^ for 1.5 h, 3.0 h, 4.5 h and 6.0 h and then cultured at 28 °C for 20 h to access the conidial germination. For fungal tolerances to different chemicals, 2 μL of the suspension (1 × 10^7^ conidia/mL) was spotted onto a 1/4 SDAY medium or 1/4 SDAY medium supplied with SDS, calcofluor white (CFW), Congo red (CR), H_2_O_2_, NaCl and sorbitol. After a 5-day cultivation, the fungal colonies were photographed, and the diameters of the colonies were recorded to calculate the relative growth inhibition rates. The fungal pathogenicity assays were determined by two methods. For topical inoculation, the suspensions of WT, ∆*MaOpy2* and CP prepared in paraffin (5 μL, 1 × 10^7^ conidia/mL) were inoculated to the fifth-instar locust. For intrahemocoel injection, the suspensions of the fungal strains prepared in ddH_2_O (1 × 10^6^ conidia/mL) were injected into locust hemocoel. The negative controls for these two methods were treated with pure paraffin oil and ddH_2_O, respectively. Every half day, the survival of the locusts was recorded.

### 2.3. Growth of Fungi in Locust Hemolymph In Vitro and Appressorium Induction on Locust Wings

To determine the appressorium formation and conidial germination, locust wings were used for analyses, as described previously [22]. Briefly, 100 μL of the conidial suspensions (1 × 10^7^ conidia/mL) was inoculated into the autoclaved locust wings and then cultured at 28 °C for different times. The conidial germination and appressorium formation were recorded under a microscope. To stain the neutral lipids, Nile red (Sigma-Aldrich, Gillingham, UK) was used [23]; then, it was photographed with fluorescence microscopy. For the fungal growth in hemolymph in vitro, the conidial suspensions (1 × 10^6^ conidia/mL, 10 μL) were inoculated into 500 μL of locust hemolymp without blood cells, which was incubated on a rotary shaker at 28 °C and 250 rpm for 4 d or 6 d. To measure the gDNA concentrations for the fungal growth in the locust blood, quantitative real-time PCR was used [24]. The detection of the conidial cell surface components was conducted as previously described [25]. The chitin of the fungal cell wall was stained with wheat germ agglutinin (WGA) (Invitrogen, Carlsbad, CA, USA). Cell wall α-1,3-glucan and β-1,3-glucan were detected using the IgMg MOPC-104E (Sigma, St, Louis, MO, USA) and β-1,3-glucan-specific antibodies (Biosupplies, Parkville, Australia) as the first antibody overnight at 37 °C, respectively, and the Alexa Fluor 488 goat anti-mouse IgM antibody (Invitrogen, Carlsbad, CA, USA) and Alexa Fluor 594 goat anti-mouse IgG antibody (Invitrogen, Carlsbad, CA, USA) were used as the secondary antibody at 37 °C for 4 h in the dark, respectively. The fluorescence of the stained conidia was detected and photographed by fluorescence microscopy (Nikon Y-TV55, Tokyo, Japan).

### 2.4. Microscopic Observation of the Conidiation Pattern and Different Expression Genes (DEGs) by RNA-seq

Fungal fresh conidia cultivated on 1/4 SDAY for 15 days were used for the conidial suspensions. An aliquot of 100 μL of the conidial suspension (1 × 10^7^ conidia/mL) of each fungal strain was spread evenly onto 1/4 SDAY plates and incubated at 28 °C. About 1 cm^2^ media was cut to detect the fungal growth by microscopic observation every 2 h under the digital light microscope (MOTIC, Xiamen, China). The RNA-sequencing was accomplished by the Beijing Genomics Institution (Wuhan, China). The DEGs were defined with a false discovery rate ≤ 0.005 and a fold change ≥ 2. The RNA-seq data were validated by quantitative reverse-transcription PCR (qRT-PCR).

### 2.5. Statistical Analysis

Tukey’s honestly significant difference (HSD) and a one-way ANOVA were applied to access the statistical differences and the phenotypic estimate, respectively.

## 3. Results

### 3.1. Characteristics of MaOpy2

**Figure 1:**
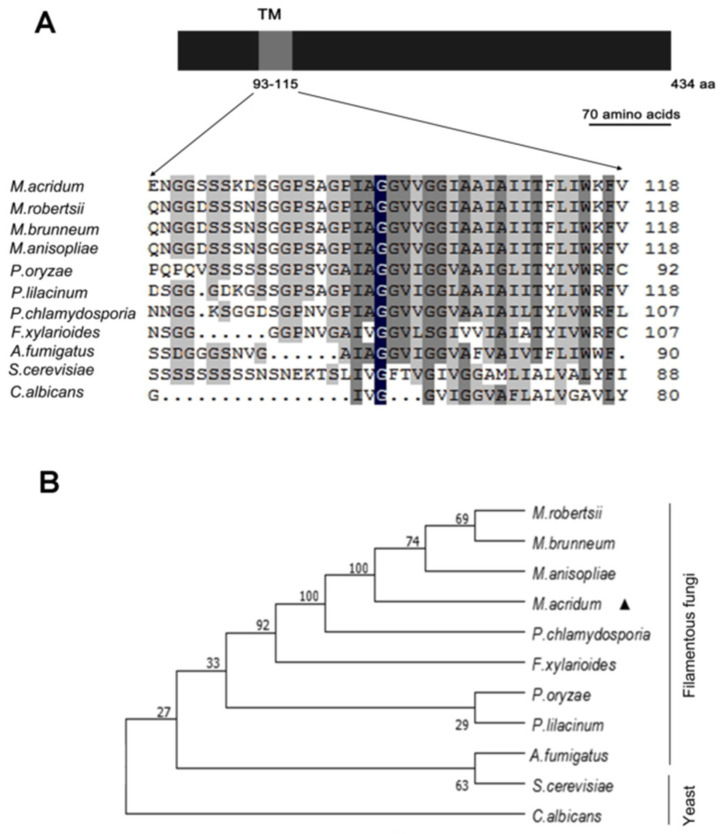
Characterization of *MaOpy2* and the phenotypic tree construction. (**A**) Conserved TM domain in Opy2. (**B**) The phenotypic tree was constructed with the Opy2 protein sequences of *Metarhizium acridum* (XP_007810157.1), *Metarhizium anisopliae* (KFG88145.1), *Metarhizium robertsii* (XP_007819189.1), *Metarhizium brunneum* (XP_014545484.1), *Pochonia chlamydosporia* (XP_018139580.1), *Fusarium xylarioides* (KAG5792966.1), *Aspergillus fumigatus* (KAF4287340.1), *Saccharomyces cerevisiae* (CAD6650216.1), *Pyricularia oryzae* (KAH8847656.1), *Candida albicans* (CAA21990.1) and *Paecilomyces lilacinus* (XP_018175377.1).

Bioinformatics analysis showed that the full length of *MaOpy2* in *M. acridum* is 1362 bp with an intron, encoding 434 amino acids. The molecular weight is 45.4 kD and the isoelectric point is 8.07. SMART (http://smart.embl.de/, accessed on 28 March 2019) was used for the domain prediction, showing that the N-terminal of *MaOpy2* has a transmembrane (TM) domain. The sequences of the TM domain were conserved in filamentous fungi, indicating that *Opy2* is a conserved membrane-anchor protein (Figure 1A). Mega v7.0 was used for the phylogenetic tree construction with the neighbor-joining method, showing that, based on the sequence homology, *MaOpy2* was well conserved in filamentous fungi (Figure 1B).

### 3.2. Disruption of MaOpy2 Decreased Fungal Tolerances to UV-B Irradiation, Heat Shock, and Cell-Wall-Perturbing Agents

**Figure 2:**
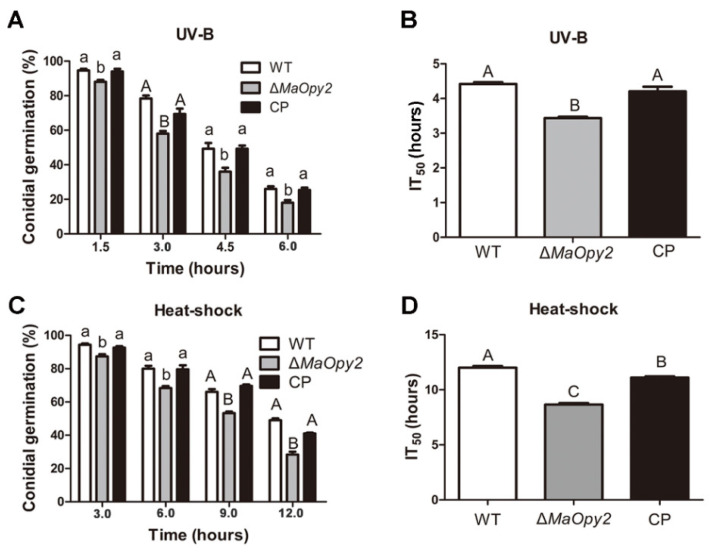
Effect of *MaOpy2* on UV-B irradiation and heat shock tolerance. (**A**) Conidial germination treated with UV-B irradiation for 1.5 h, 3.0 h, 4.5 h and 6.0 h. (**B**) The IT_50_ of UV-B irradiation. (**C**) Germination rates treated with heat shock for 3.0 h, 6.0 h, 9.0 h and 12.0 h. (**D**) IT_50_ of heat shock. A & B & C and a & b presented the significant difference at *p* < 0.01 and *p* < 0.05, respectively.

**Figure 3:**
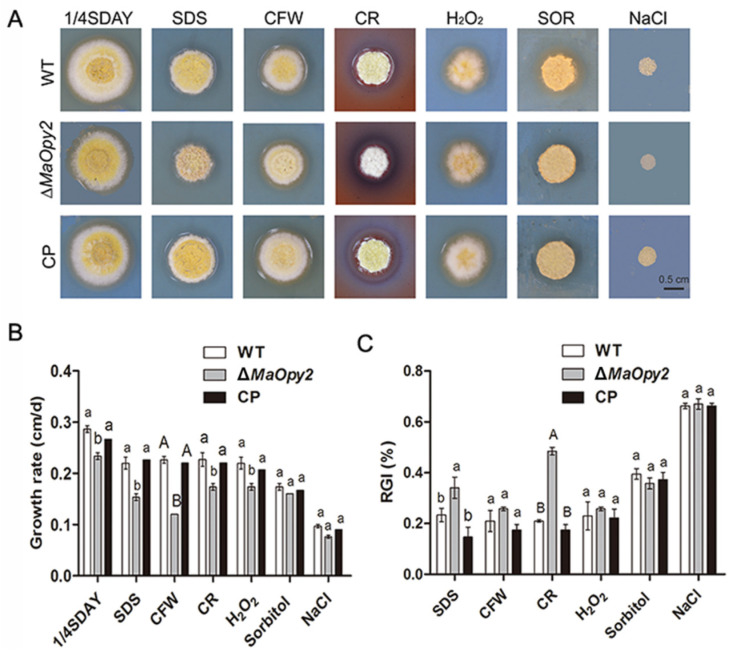
Tolerance to chemicals. (**A**) Colonies of different strains on the 1/4 SDAY medium or 1/4 SDAY medium supplied with different chemical compounds for 5 days. (**B**) Fungal growth rate on the different medium for the 5-day cultivation. (**C**) Relative growth inhibition by different chemicals. A & B and a & b presented the significant difference at *p* < 0.01 and *p* < 0.05, respectively.

To reveal the roles of *MaOpy2*, the strategies of homologous recombination were used to construct the *MaOpy2* deletion strain (∆*MaOpy2*) and the complementation strain (CP) (Figure S1A,B). Southern blotting was used to confirm the successful transformants (Figure S2C). Conidial germination assays were conducted after the treatment with UV-B irradiation and heat shock. The UV-B tolerance in ∆*MaOpy2* was reduced significantly (Figure 2A). The half-inhibition times of germination (IT_50_s) by UV-B irradiation for the WT and CP strains were 4.42 ± 0.88 h and 4.21 ± 0.48 h, respectively, while the IT_50_ of ∆*MaOpy2* was 3.42 ± 0.29 h (Figure 2B; *p* < 0.01). Additionally, the tolerance of ∆*MaOpy2* to heat shock was also significantly impaired (Figure 2C; *p* < 0.01). The IT_50_ of ∆*MaOpy2* was 8.65 ± 0.76 h—significantly decreased compared to that of the WT (12.20 ± 0.89 h) and CP strains (11.10 ± 0.97 h, Figure 2D; *p* < 0.01). The spot assays revealed that the disruption of *MaOpy2* rendered the fungi more sensitive to the cell-wall-disturbing agents SDS and CR; however, the deletion of *MaOpy2* did not change the fungal tolerances to oxidative (H_2_O_2_) and hyperosmotic stresses (NaCl and Sorbitol) (Figure 3).

### 3.3. Deletion of MaOpy2 Impaired the Insect Cuticle Penetration of M. acridum

**Figure 4:**
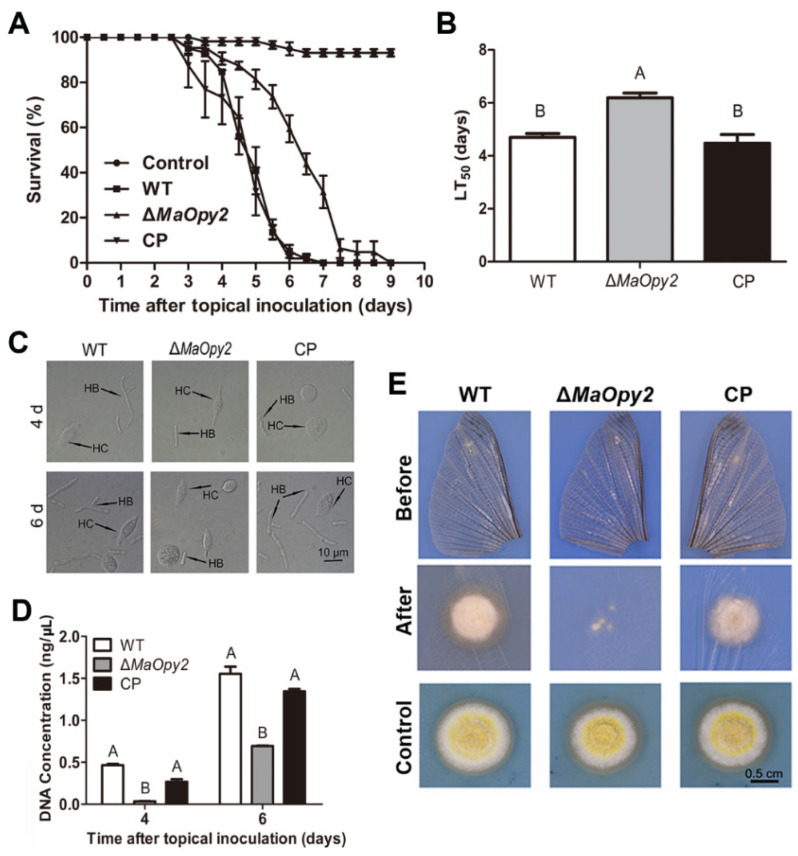
Topical inoculation assays. (**A**) Survivals of locusts by topical inoculation. (**B**) LT_50_s of fungal strains for topical inoculation. (**C**) Fungal growth inside insect hemolymph by topical inoculation for 4 d or 6 d. (**D**) Quantification of fungal gDNA through topical inoculation. (**E**) Penetration assays. A & B presented the significant difference at *p* < 0.01.

**Figure 5:**
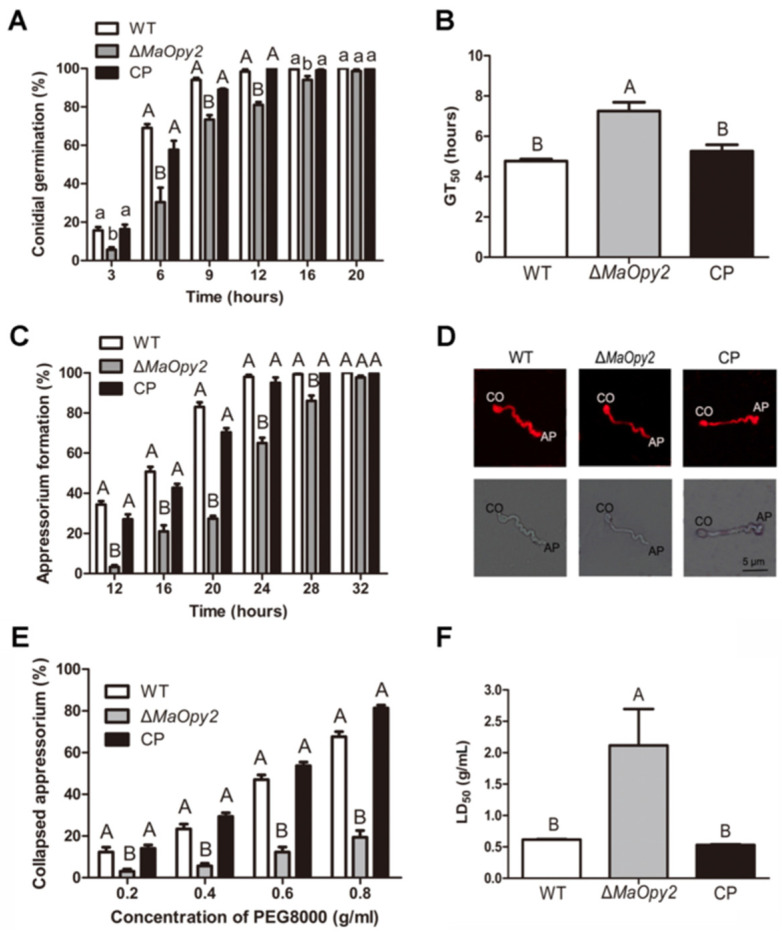
Appressorium assays. (**A**) Germination of conidia on the locust wings of fungal strains. (**B**) The GT_50_s of different strains on locust wings. (**C**) Appressorium formation of different fungal strains. (**D**) Lipid distribution in appressoria stained with Nile red. (**E**) Collapsed appressorium treated with different concentrations of PEG 8000. (**F**) LT_50_s of PEG 8000 for fungal strains. A & B and a & b presented the significant difference at *p* < 0.01 and *p* < 0.05, respectively.

For topical inoculation, the disruption of *MaOpy2* decreased fungal pathogenicity compared to WT and CP. The locusts all died at day 7 when inoculated with WT and CP; however, when infected with ∆*MaOpy2*, the locusts died at day 9 (Figure 4A). In addition, the ∆*MaOpy2* strain exhibited significantly longer LT_50_ (6.19 ± 0.20 d), while the LT_50_s of the WT and CP strains were 4.70 ± 0.11 d and 4.48 ± 0.26 d, respectively (*p* < 0.01; Figure 4B). The fungal infection assays showed that, after the inoculation of the locusts for 4 days and 6 days, there were less hyphae bodies in the locusts incubated with ∆*MaOpy2* than those in the other strains (Figure 4C). After 6 d post-inoculation, the fungal gDNA concentrations were decreased in ∆*MaOpy2* (0.69 ± 0.03 ng/μL) when compared to those in WT (1.55 ± 0.11 ng/μL) and CP (1.34 ± 0.1 ng/μL, *p* < 0.05; Figure 4D). To further explore whether *MaOpy2* deletion affects fungal penetration, a spot assay was performed on the locusts’ hind wings, and the results indicated that the fungal colony of ∆*MaOpy2* was smaller than that of the other strains (Figure 4E). Furthermore, conidial germination on the locust wings and appressorium formation accesses were both conducted. When cultured for 6 h, the conidia germination rate of ∆*MaOpy2* was 33.3%—significantly lower than that of the WT (69.0%) and CP (57.6%) strains (*p* < 0.01; Figure 5A). At 20 h, the conidial germination rates of all the strains reached the same level (Figure 5A). The GT_50_ of ∆*MaOpy2* (7.25 ± 0.46 h) was significantly decreased compared with that of WT (4.77 ± 0.54) and CP (5.26 ± 0.54 h, *p* < 0.01; Figure 5B). In addition, compared with that of the WT (34.3%) and CP (27.0%) strains, the appressorium formation of ∆*MaOpy2* (3.3%) was significantly reduced when cultured for 12 h (Figure 5C). In addition, Nile red was used to measure the pressure in appressorium. However, it was revealed that ∆*MaOpy2* presented a weaker fluorescence intensity compared to that presented by WT and CP (Figure 5D), suggesting that the content of neutral lipids in ∆*MaOpy2* was reduced. To investigate the turgor pressure in the fungal appressoria, different concentrations of PEG-8000 were used, and the results revealed that the appressoria of ∆*MaOpy2* collapsed more difficultly than those of WT and CP (Figure 5E,F), suggesting an increased turgor pressure in ∆*MaOpy2*. The results above indicate that the *MaOpy2* disruption impaired the pre-penetration and penetration of insect cuticle in *M. acridum*.

### 3.4. Deletion of MaOpy2 Enhanced the Colonized Ability of M. acridum in Locust Hemolymph

**Figure 6:**
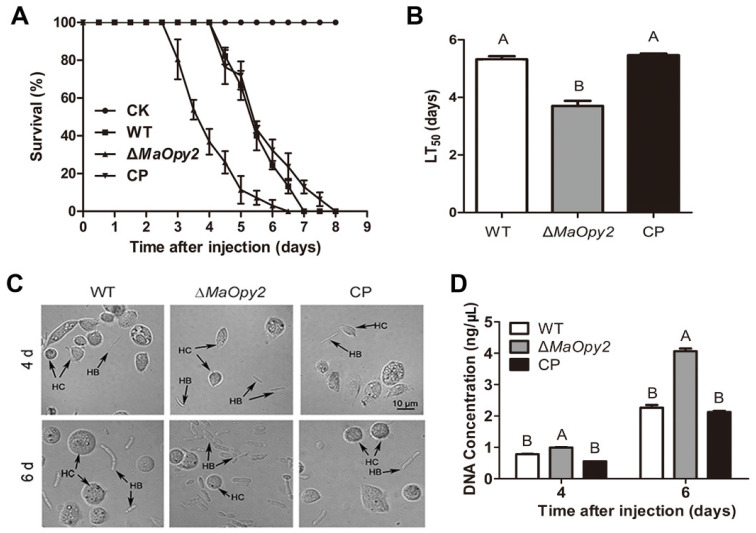
Injection assays. (**A**) Survivals of locusts by injection. (**B**) LT_50_s of fungal strains for injection. (**C**) Fungal growth inside insect hemolymph by injection inoculation for 4 d or 6 d. (**D**) Quantification of fungal strains in locust hemolymph. A & B presented the significant difference at *p* < 0.01.

**Figure 7:**
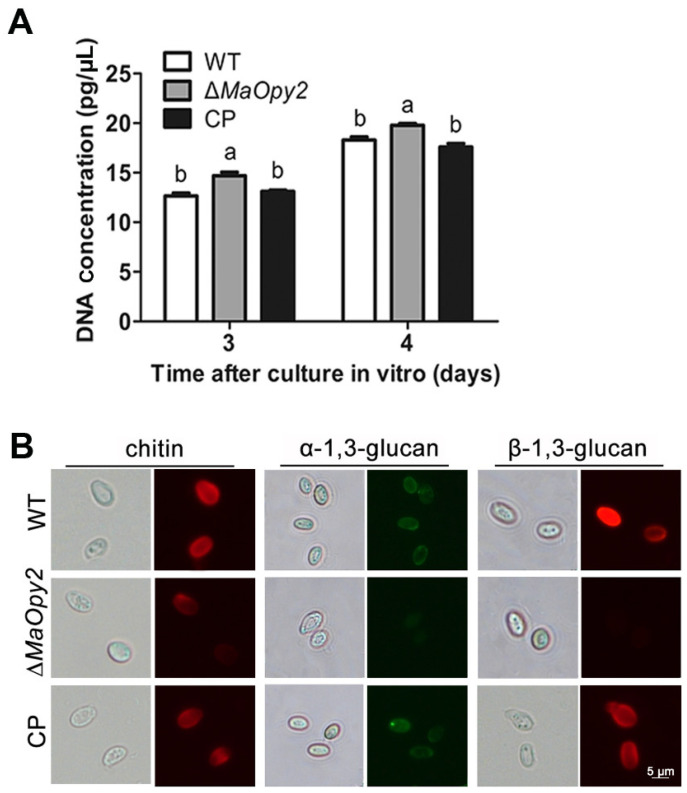
Detection of fungal growth and conidial cell surface components. (**A**) Concentration of fungal gDNA in host hemolymph without blood cells in vitro. (**B**) Detection of conidial cell surface components with labeled lectins and antibodies. a & b presented the significant difference at *p* < 0.05.

For intrahemocoel injection, however, the deletion of *MaOpy2* increased the fungal virulence significantly. The LT_50_ of ∆*MaOpy2* was 3.7 ± 0.16 d—significantly decreased compared with that of the WT (5.32 ± 0.33 d) and CP (5.46 ± 0.34 d) strains (*p* < 0.01; Figure 6A,B). Consistently, the treatment with ∆*MaOpy2* led to a higher fungal growth after 6 d injection (*p* < 0.05; Figure 6C,D). Moreover, the fungal growth in the locust hemolymph without hemocytes in vitro was strengthened, and after 3 d inoculation, the gDNA concentrations of WT, ∆*MaOpy2* and CP were 12.66 ± 0.94 pg/μL, 14.7 ± 1.10 pg/μL, and 13.11 ± 0.43 pg/μL, respectively, (*p* < 0.05; Figure 7A), showing that the deletion of *MaOpy2* promoted fungal growth in hemolymph in vitro. Moreover, fluorescent staining revealed that the conidia of ∆*MaOpy2* showed dramatically decreased contents of chitin, α-1,3-glucan and β-1,3-glucan (Figure 7B). These results indicate that the *MaOpy2* deletion enhanced the colonized ability of *M. acridum* in locust hemolymph.

### 3.5. Deletion of MaOpy2 Increased Fungal Conidial Yield by Shifting the Conidiation Pattern

**Figure 8:**
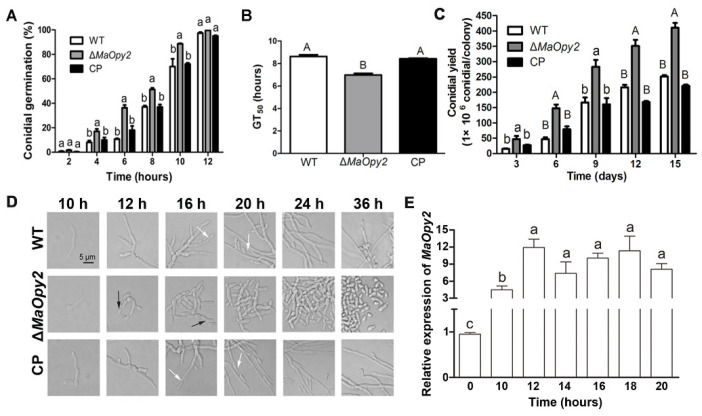
Deletion of *MaOpy2* increases the conidial yield by shifting the conidiation pattern. (**A**) Conidial germination of fungal strains on a 1/4 SDAY medium. (**B**) Half germination time (GT_50_) of fungal strains. (**C**) Conidial yield of fungal strains on a 1/4 SDAY medium. (**D**) Deletion of *MaOpy2* shifts the conidiation pattern. Microscopic observation of the conidiation pattern of each strain grown on 1/4 SDAY for different times; white arrows and black arrows represent normal conidiation and microcirculation conidiation, respectively. (**E**) Expression levels of *MaOpy2* at different time points. A & B and a & b & c presented the significant difference at *p* < 0.01 and *p* < 0.05, respectively.

The fungal growth was examined by the conidial germination and conidial yield, and the results showed that the disruption of *MaOpy2* accelerated conidial germination. Compared with those of WT (8.64 ± 0.88 h) and CP (8.44 ± 0.70 h), the GT_50_ of ∆*MaOpy2* was significantly decreased (6.88 ± 0.66 h, *p* < 0.01; Figure 8A,B). The deletion of *MaOpy2* increased the conidial yield in 1/4 SDAY by one time (Figure 8C). To gain a deeper insight into the roles of *MaOpy2* in the conidial yield, the conidiation process was microscopically observed. At 12 h, ∆*MaOpy2* began to generate conidia at the apex of the hypha (black arrows in Figure 8D); however, the WT and CP strains both grew with long hyphae. At 16 h, ∆*MaOpy2* began to exhibit the typical microcycle conidiation, whereas the WT and CP strains began to form conidiophores at the apex of the hyphae (white arrows in Figure 8D). At 36 h, ∆*MaOpy2* generated a great number of conidia; however, the WT and CP strains still grew with long hyphae and exhibited normal conidiation (Figure 8D). The *MaOpy2* expression levels were determined by qRT-PCR during the conidiation pattern shift. The results showed that the expression level of *MaOpy2* reached its peak at 12 h and was about threefold greater than that at 10 h (Figure 8E). The results above indicate that *MaOpy2* governed the conidiation pattern shift in *M. acridum*.

### 3.6. Identification of the DEGs Regulated by MaOpy2 during a Conidiation Pattern Shift Using RNA-seq

**Figure 9:**
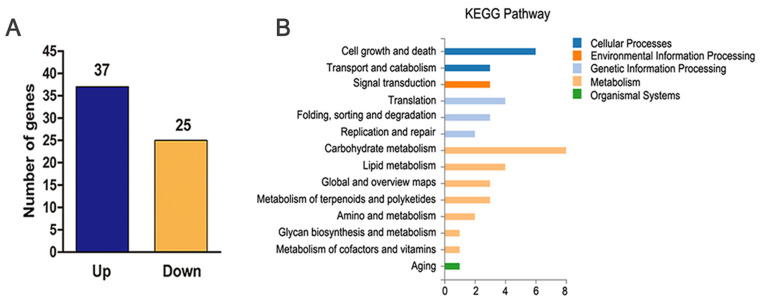
Analyses of differentially expressed genes. (**A**) Numbers of differentially expressed genes. (**B**) GO annotation of differentially expressed genes.

To gain an insight into the role of *MaOpy2* in shifting the conidiation pattern, RNA-seq was adopted to identify the genes regulated by *MaOpy2*. Combined with the morphology that the strains exhibited (Figure 8D) and the *MaOpy2* expression (Figure 8E), the total RNA from WT and ∆*MaOpy2* on 1/4 SDAY at 12 h were isolated for sequencing. Among the 62 DEGs, 37 were upregulated and 25 were downregulated (Figure 9A). Thirty-two DEGs were annotated as hypothetical proteins (Table S2). Sixteen DEGs, including eight upregulated and eight downregulated genes, were used for the qRT-PCR analysis to validate the reliability of the RNA-seq data. The results revealed that, compared to the RNA-seq data, all the selected genes displayed similar expression patterns (Figure S2), indicating that the RNA-seq data were reliable. Through gene ontology (GO) annotation, the DEGs were classified into catalytic activity, binding, membrane, membrane part, metabolic processes and cellular processes (Figure 9B). The genes involved in the conidiation pattern shift are listed in Table S3, such as a downregulated gene for cation-transporting ATPase 4 (MAC_09130) and two upregulated genes for the pantothenate transporter (MAC_06827) and putative endoglucanase (MAC_02571), which were related to hyphal growth and conidiation, demonstrating that *MaOpy2* regulates the shift of the conidiation pattern.

## 4. Discussion

*Opy2* is a conserved membrane-anchor protein upstream of the HOG-MAPK signaling pathway. In this study, the homolog recombination strategy was used to obtain the *MaOpy2* deletion and complementation transformants, and the functions of *MaOpy2* were systematically analyzed. The deletion of *MaOpy2* significantly impaired the fungal tolerance to UV-B irradiation, heat shock and cell-wall-interfering compounds. Interestingly, the deletion of *MaOpy2* decreased the fungal virulence through topical inoculation but enhanced the fungal virulence by injection, suggesting a unique role of *MaOpy2* in fungal pathogenicity. More importantly, the disruption of *MaOpy2* increased the conidial yield by shifting the conidiation pattern from normal conidiation to microcycle conidiation, and RNA-seq was used to explore the possible mechanism in the *MaOpy2* regulation of the conidiation pattern shift.

For entomopathogenic fungi, stress tolerance is a vital factor to survive and infect hosts [2]. Our results gave the first insights into the functions of *Opy2* in UV and heat shock tolerance in entomopathogenic fungi, which have never been reported before. It was revealed that the deletion of *MaOpy2* significantly decreased the fungal tolerance to UV-B irradiation and heat shock. Based on the RNA-seq data, some DEGs related to the cell wall reorganization were found to be differentially expressed. MAC_01372, a gene for the Glycosyl hydrolase family 16 protein, which was involved in the process of β-1,3-glucan degradation [26], was upregulated in ∆*MaOpy2*. MAC_04906, a gene for the putative cell wall glycosyl hydrolase Dfg5, was downregulated. In *S. cerevisiae*, Dfg5 was an essential component of the cell wall and was vital for cell growth [26]. A gene for the serine/threonine-protein kinase GIN4 (MAC_00865) was found to be upregulated. In *Fusarium graminearum,* the disruption of a GIN4-like protein kinase gene increased the fungal tolerance to cell wall stress [27], suggesting a possibility that the Gin4 regulation of the fungal tolerance to cell wall stress by *MaOpy2* functioned differentially in *M. acridum*. In addition, ∆*MaOpy2* exhibited a higher susceptibility to the cell-wall-disturbing agent CR, which was consistent with that in *C. albicans* [17]. However, *MaOpy2* was a dispensable fungal adaption to high osmolarity or oxidative stress, which was in accordance with that in *C. albicans* [17] but contrast with that in *S. cerevisiae*.

It was indicated that *MaOpy2* played a totally distinct role in fungal pathogenicity by two different methods. On one hand, the fungal virulence was significantly attenuated by topical inoculation by impairing the appressorium formation. Our results are well in accordance with the results in another fungus. In *M. robertsii*, both a low *MrOpy2* protein level and the deletion of *MrOpy2* could impair appressorium formation and thereby reduce fungal pathogenicity to hosts [18]. However, it was found that the deletion of *MaOpy2* increased the appressorium pressure, in contrast with the result in *M. oryzae*, where *MoOpy2* deletion reduced the appressorium pressure [19]. A previous study has shown that there are similar cases in which increased turgor pressure led to decreased virulence [21,28]. Intracellular glycerol is essential for the appressorium to generate huge pressure for the successful penetration of the host cuticle [29,30]. The deletion of *MaOpy2* impaired fungal virulence, leading to a lower lipid content and increased turgor pressure, suggesting that the decreased virulence may result from the weakened pre-penetration and penetration process. In *C. albicans*, a *CaOpy2* mutant also displayed a significantly reduced virulence in the *Galleria mellonella* model [17].

On the other hand, the deletion of *MaOpy2* significantly enhanced the fungal virulence through injection, which had never been reported. Our results proved that the increased virulence was mainly due to the enhanced fungal colonization inside insects. The host innate immune system could recognize the specific components of the fungal cell wall [31]. Compared to WT and CP, the distribution of α-1,3-glucan, chitin and β-1,3-glucan on the conidial surface of ∆*MaOpy2* was obviously decreased, which contributed to the increased fungal virulence by intrahemocoel injection.

More importantly, *MaOpy2* deletion could increase the fungal conidial yield by shifting the conidiation pattern from normal conidiation to microcycle conidiation. Three membrane proteins, including *Opy2*, *Sho1* and *Msb2*, comprise the SHO1-branch of the HOG-MAPK signaling pathway. A previous study has shown that the deletion of *MaSho1* could also shift the conidiation pattern to microcycle conidiation in *M. acridum* [32]. However, *MaHog1* made no contribution to shifting the conidiation pattern [32], suggesting that *MaOpy2* may function together with *MaSho1* during the conidiation pattern shift, which is independent of the conserved HOG-MAPK signaling pathway.

To reveal the mechanism of *MaOpy2* regulating the conidiation pattern shift, RNA-seq was performed to screen some possible genes that played important roles during the conidiation pattern shift. Among the DEGs, some genes related to the hyphae growth, cell cycle and conidiation were found to be differentially expressed. A gene for the pantothenate transporter (MAC_06827) was upregulated in ∆*MaOpy2*. A previous study revealed that a pantothenate transporter gene disruption mutant could not form spores [33]. In some fungi, the serine/threonine-protein kinase Gin4 was important for the septin organization and cell cycle [34,35,36]. The inactivation of Gin4 led to a prolonged mitotic delay [36]. In the RNA-seq data, the gene for the serine/threonine-protein kinase Gin4 (MAC_00865) was upregulated, which may accelerate the process of cell division, suggesting the possibility that *MaOpy2* may regulate the expression of Gin4 to shift the conidiation pattern, thus promoting the conidiation—but this needs to be further explored.

## Data Availability

The raw data from the RNA-seq have been deposited into the NCBI BioProject database under the accession number PRJNA833156.

## Supplementary Materials

The following supporting information can be downloaded at: https://www.mdpi.com/article/10.3390/jof8060587/s1, Figure S1: Disruption and complementation of MaOpy2. (A) Schematic illustration of MaOpy2 disruption. (B) Design of the MaOpy2 complementation vector. (C) Southern blotting. Genomic DNA from WT, ∆MaOpy2 and CP were digested with Pvu I and Kpn I. WT: wild type; ∆MaOpy2: the MaOpy2 disruption mutant; CP: complemented mutant. Figure S2. Validation of the RNA-seq data by qRT-PCR. Table S1: Primers used in this study. Table S2: Differentially expressed genes. Table S3: DEGs involved in stress tolerance and conidiation pattern shift [26,27,33,34,35,36].

## References

[1] Clarkson J.M., Charnley A.K. (1996). New insights into the mechanisms of fungal pathogenesis in insects. Trends. Microbiol..

[2] Ortiz-Urquiza A., Luo Z.B., Keyhani N.O. (2015). Improving mycoinsecticides for insect biological control. Appl. Microbiol. Biotechnol..

[3] Brodeur J. (2012). Host specificity in biological control: Insights from opportunistic pathogens. Evol. Appl..

[4] Zarrin M., Rahdar M., Gholamian A. (2015). Biological Control of the Nematode Infective larvae of Trichostrongylidae Family With Filamentous Fungi. Jundishapur. J. Microbiol..

[5] Rangel D.E., Braga G.U., Anderson A.J., Roberts D.W. (2005). Influence of growth environment on tolerance to UV-B radiation, germination speed, and morphology of Metarhizium anisopliae var. acridum conidia. J. Invertebr. Pathol..

[6] Thomas M.B., Read A.F. (2007). Can fungal biopesticides control malaria?. Nat. Rev. Microbiol..

[7] Thompson S.R., Brandenburg R.L., Arends J.J. (2006). Impact of moisture and UV degradation on Beauveria bassiana (Balsamo) Vuillemin conidial viability in turfgrass. Biol. Control.

[8] Ferron P. (1978). Biological Control of Insect Pests by Entomogenous Fungi. Annu. Rev. Entomol..

[9] Hanlin R. (1994). Microcycle conidiation—A review. Mycoscience.

[10] Jung B., Kim S., Lee J. (2014). Microcyle conidiation in filamentous fungi. Mycobiology.

[11] Anderson J.G., Smith J.E. (1971). The production of conidiophores and conidia by newly germinated conidia of Aspergillus niger (microcycle conidiation). J. Gen. Microbiol..

[12] Pažout J., Schröder P. (1988). Microcycle conidiation in submerged cultures of Penicillium cyclopium attained without temperature changes. J. Gen. Microbiol..

[13] Vezina C., Singh K., Sehgal S.N. (1965). Sporulation of filamentous fungi in submerged culture. Mycologia.

[14] Bosch A., Yantorno O. (1999). Microcycle conidiation in the entomopathogenic fungus Beauveria bassiana bals. (vuill.). Process Biochem..

[15] Zhang S.Z., Peng G.X., Xia Y.X. (2010). Microcycle conidiation and the conidial properties in the entomopathogenic fungus Metarhizium acridum on agar medium. Biocontrol Sci. Techn..

[16] Wu C., Jansen G., Zhang J.C., Thomas D.Y., Whiteway M. (2006). Adaptor protein Ste50p links the Ste11p MEKK to the HOG pathway through plasma membrane association. Genes Dev..

[17] Herrero de Dios C., Roman E., Diez C., Alonso-Monge R., Pla J. (2013). The transmembrane protein Opy2 mediates activation of the Cek1 MAP kinase in Candida albicans. Fungal Genet. Biol..

[18] Guo N., Qian Y., Zhang Q.Q., Chen X.X., Zeng G.H., Zhang X., Mi W.B., St Leger R., Fang W.G. (2017). Alternative transcription start site selection in Mr-OPY2 controls lifestyle transitions. Nat. Commun..

[19] Cai Y.Y., Wang J.Y., Wu X.Y., Liang S., Zhu X.M., Li L., Lu J.P., Liu X.H., Lin F.C. (2022). MoOpy2 is essential for fungal development, pathogenicity, and autophagy in Magnaporthe oryzae. Environ. Microbiol..

[20] Wen Z.Q., Tian H.T., Xia Y.X., Jin K. (2020). MaPmt1, a protein O-mannosyltransferase, contributes to virulence through governing the appressorium turgor pressure in Metarhizium acridum. Fungal Genet. Biol..

[21] Wen Z.Q., Xia Y.X., Jin K. (2022). MaSln1, a Conserved Histidine Protein Kinase, Contributes to Conidiation Pattern Shift Independent of the MAPK Pathway in Metarhizium acridum. Microbiol. Spectr..

[22] He M., Xia Y.X. (2009). Construction and analysis of a normalized cDNA library from Metarhizium anisopliae var. acridum germinating and differentiating on Locusta migratoria wings. FEMS Microbiol. Lett..

[23] Wang C.S., St Leger R.J. (2007). The Metarhizium anisopliae perilipin homolog MPL1 regulates lipid metabolism, appressorial turgor pressure, and virulence. J. Biol. Chem..

[24] Zhang Q.P., Peng G.X., Wang Z.K., Yin Y.P., Xia Y.X. (2005). Detection of Metarhizium anisopliae rDNA in haemalymph of infected adult locust by real-time fluorescence quantitative PCR. Chin. Prog. Biotech..

[25] Zhang J.J., Jiang H., Du Y.R., Keyhani N.O., Xia Y.X., Jin K. (2019). Members of chitin synthase family in Metarhizium acridum differentially affect fungal growth, stress tolerances, cell wall integrity and virulence. PLoS Pathog..

[26] Kitagaki H., Wu H., Shimoi H., Ito K. (2002). Two homologous genes, DCW1 (YKL046c) and DFG5, are essential for cell growth and encode glycosylphosphatidylinositol (GPI)-anchored membrane proteins required for cell wall biogenesis in Saccharomyces cerevisiae. Mol. Microbiol..

[27] Yu D., Zhang S.J., Li X.P., Xu J.R., Schultzhaus Z., Jin Q.J. (2017). A Gin4-Like Protein Kinase GIL1 Involvement in Hyphal Growth, Asexual Development, and Pathogenesis in Fusarium graminearum. Int. J. Mol. Sci..

[28] Ryder L.S., Dagdas Y.F., Kershaw M.J., Venkataraman C., Madzvamuse A., Yan X., Cruz-Mireles N., Soanes D.M., Oses-Ruiz M., Styles V. (2019). A sensor kinase controls turgor-driven plant infection by the rice blast fungus. Nature.

[29] Cross A.R., Jones O.T. (1986). The effect of the inhibitor diphenylene iodonium on the superoxide-generating system of neutrophils. Specific labelling of a component polypeptide of the oxidase. Biochem. J..

[30] Howard R.J., Ferrari M.A., Roach D.H., Money N.P. (1991). Penetration of hard substrates by a fungus employing enormous turgor pressures. Proc. Natl. Acad. Sci. USA.

[31] Latgé J.P. (2007). The cell wall: A carbohydrate armour for the fungal cell. Mol. Microbiol..

[32] Zhao T.T., Wen Z.Q., Xia Y.X., Jin K. (2020). The transmembrane protein MaSho1 negatively regulates conidial yield by shifting the conidiation pattern in Metarhizium acridum. Appl. Microbiol. Biotechnol..

[33] Hart R.J., Lawres L., Fritzen E., Ben Mamoun C., Aly A.S. (2014). Plasmodium yoelii vitamin B5 pantothenate transporter candidate is essential for parasite transmission to themosquito. Sci. Rep..

[34] Li C.R., Yong J., Wang Y.M., Wang Y. (2012). CDK regulates septin organization through cell-cycle-dependent phosphorylation of the Nim1-related kinase Gin4. J. Cell Sci..

[35] Meitinger F., Pereira G. (2017). The septin-associated kinase Gin4 recruits Gps1 to the site of cell division. Mol. Biol. Cell.

[36] Jasani A., Huynh T., Kellogg D.R. (2020). Growth-Dependent Activation of Protein Kinases Suggests a Mechanism for Measuring Cell Growth. Genetics.

