# Supplementary material for: MaOpy2, a Transmembrane Protein, Is Involved in Stress Tolerances and Pathogenicity and Negatively Regulates Conidial Yield by Shifting the Conidiation Pattern in Metarhizium acridum
Source: J Fungi (Basel). 2022 May 30;8(6):587. doi: 10.3390/jof8060587 (PMC9225090; doi:10.3390/jof8060587)
Supplement: Supplementary file 1 [file jof-08-00587-s001.zip › Supplementary Material/jof-1729089- Supplementary-Figure S1,S2.pdf]

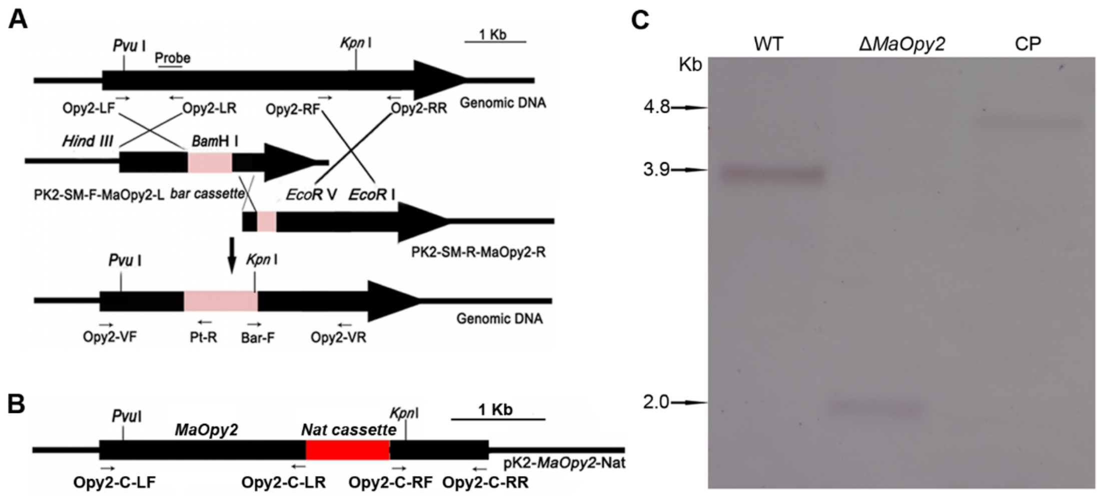

Figure S1. Disruption and complementation of *MaOpy2*.

(A) Schematic illustration of *MaOpy2* disruption. (B) Design of the *MaOpy2* complementation vector. (C) Southern blotting. Genomic DNA from WT, Δ*MaOpy2* and CP were digested with *Pvu* I and *Kpn* I. WT: wild type; Δ*MaOpy2*: the *MaOpy2* disruption mutant; CP: complemented mutant.

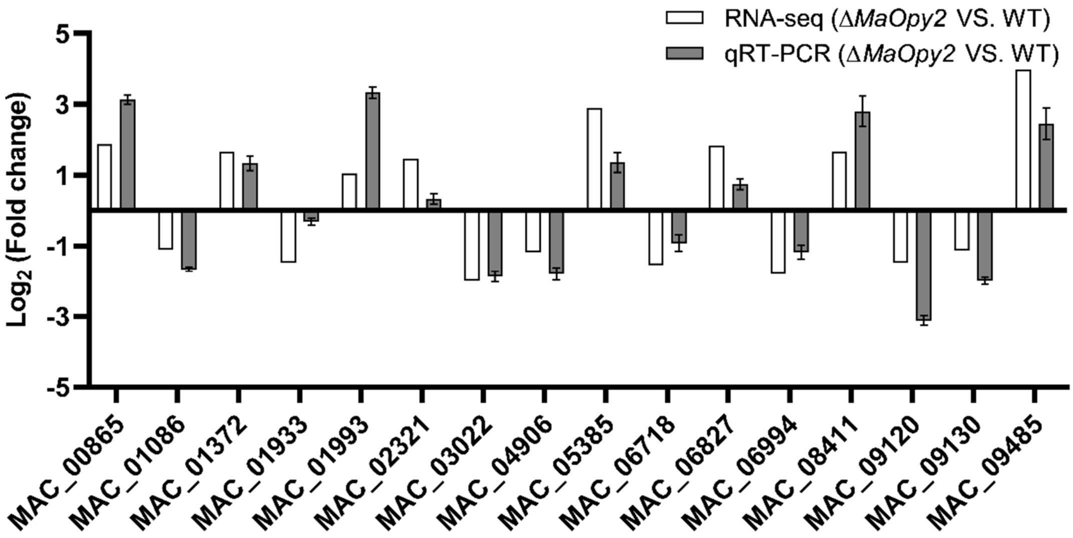

Figure S2. Validation of the RNA-seq data by qRT-PCR.
