# Supplementary material for: MaOpy2, a Transmembrane Protein, Is Involved in Stress Tolerances and Pathogenicity and Negatively Regulates Conidial Yield by Shifting the Conidiation Pattern in Metarhizium acridum
Source: J Fungi (Basel). 2022 May 30;8(6):587. doi: 10.3390/jof8060587 (PMC9225090; doi:10.3390/jof8060587)
Supplement: Supplementary file 1 [file jof-08-00587-s001.zip › Supplementary Material/Table S3 DEGs involved in stress tolerance and conidiation pattern shift.pdf]

Table S3 DEGs involved in stress tolerance and conidiation pattern shift.

Gene ID	log2 (Δ*MaOpy2*/WT)	Q value (Δ*MaOpy2*/WT)	Description	Reference
MAC_00865	1.871183843	8.54E-13	serine/threonine-protein kinase GIN4	[27,34–36]
MAC_01372	1.659528474	1.33E-18	Glycosyl hydrolase family 16 protein	[26]
MAC_04906	-1.18489314	0.00882945	cell wall glycosyl hydrolase Dfg5, putative	[26]
MAC_06827	1.832997724	0.005786526	pantothenate transporter	[33]
